# Malignant adnexal tumors of the skin: a single institution experience

**DOI:** 10.1186/s12957-018-1401-y

**Published:** 2018-05-30

**Authors:** Tolutope Oyasiji, Wei Tan, John Kane, Joseph Skitzki, Valerie Francescutti, Kilian Salerno, Nikhil I. Khushalani

**Affiliations:** 1 Department of Surgical Oncology, Roswell Park Cancer Institute, Elm and Carlton Streets, Buffalo, NY 14263 USA; 2 Department of Surgical Oncology, Barbara Ann Karmanos Cancer Institute at McLaren Flint, 4100 Beecher Road, Flint, MI 48532 USA

**Keywords:** Malignant, Adnexal, Tumors, Skin, Survival, Treatment

## Abstract

**Background:**

Malignant adnexal tumors of the skin (MATS) are rare. We aimed to measure the survival of patients with MATS and identify predictors of improved survival.

**Methods:**

A retrospective review of MATS treated at our institution from 1990 to 2012.

**Results:**

There were 50 patients within the time period. Median age was 59.5 years (range 22–95); primary site was the head and neck (52%); most common histologic subtypes were skin appendage carcinoma (20%) and eccrine adenocarcinoma (20%); and the vast majority were T1 (44%). Most patients (98%) underwent surgical treatment. Chemotherapy and radiation were administered to 8 and 14% of patients, respectively. Recurrence rate was 12%. Median OS was 158 months (95% CI, 52–255). OS and recurrence-free survival at 5 years were 62.4 and 47.4% and at 10 years 56.7 and 41.5%, respectively. Five-year and 10-year disease-specific survival (DSS) was 62.9%. Age > 60 years was an unfavorable predictor of OS (HR 12.9, *P* < .0008) and recurrence-free survival (RFS) (HR 12.53, *P* < .0003). Nodal metastasis was a negative predictor of RFS (HR 2.37, *P* < 0.04) and DSS (HR 7.2, *P* < 0.03) while treatment with chemotherapy was predictive of poor DSS (HR 14.21, *P* < 0.03).

**Conclusions:**

Younger patients had better OS and RFS. Absence of nodal metastasis translated to better RFS and DSS. Lymph node basin staging is worth considering in the workup and treatment.

## Background

Malignant adnexal tumors of the skin (MATS) are a heterogeneous group of rare tumors without consensus on management guidelines. There are different histologic entities based on varying differentiation from eccrine, apocrine, sebaceous, sweat duct, or ceruminous glands within the skin or follicular cells [1]. They vary in behavior and malignant potential and pose a diagnostic challenge for pathologists and surgeons [2]. Paucity of scientific information on these tumors is reflected by the fact that categorization under the WHO classification of skin carcinomas was performed only in 2005 [3]. The AJCC staging for non-melanoma and non-Merkel cell skin tumors is applied to this group of tumors. The age-adjusted incidence rate for MATS is 5.1 per one million person-years. The incidence rate among men is statistically significantly higher than women (6.3 vs 4.2, respectively; male to female incidence rate ratio is 1.51; *P* < .001). The incidence rates for these tumors have increased by as much as 150% in the last three decades making it imperative that we expand our understanding of these tumors to make informed decisions regarding prognosis and treatment [4]. With this in mind, we sought to define our experience in MATS.

## Methods

A retrospective review of all MATS treated at the Roswell Park Cancer Institute between January 1, 1990, and August 31, 2012, was carried out. An institutional review board approval was obtained for the study. These patients were identified through the Institute’s tumor registry. Relevant demographic, clinical, staging, pathologic, and outcome data were obtained for each patient. Adult patients aged 18 years and above with histologic diagnosis of malignant adnexal tumors of the skin were included in the study. Patients with concurrent diagnosis of squamous cell carcinoma, basal cell carcinoma, and melanoma were excluded.

Descriptive characteristics like frequencies were computed for categorical variables like gender, histologic diagnosis, primary site of disease, and type of surgical treatment, while numeric variables were summarized using mean with standard deviation, median, and range.

**Fig. 1:**
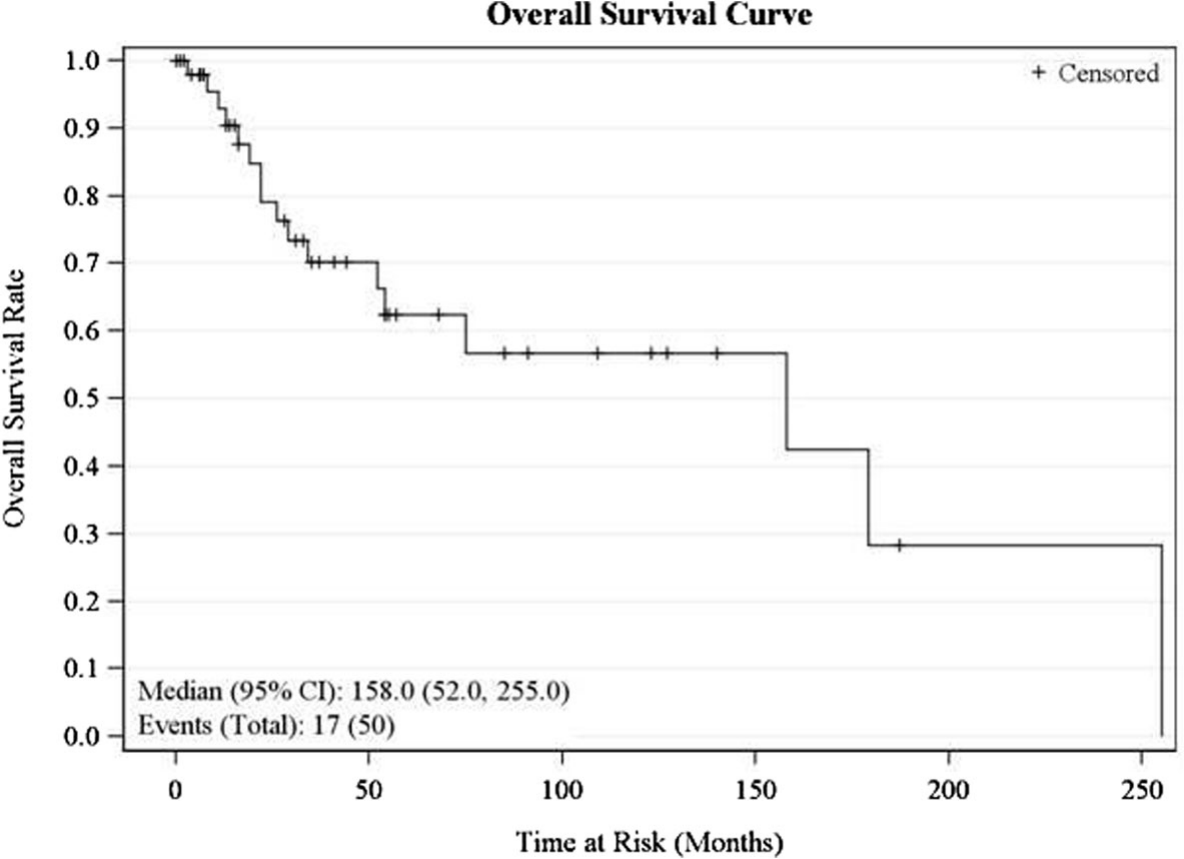
Overall survival curve

**Fig. 2:**
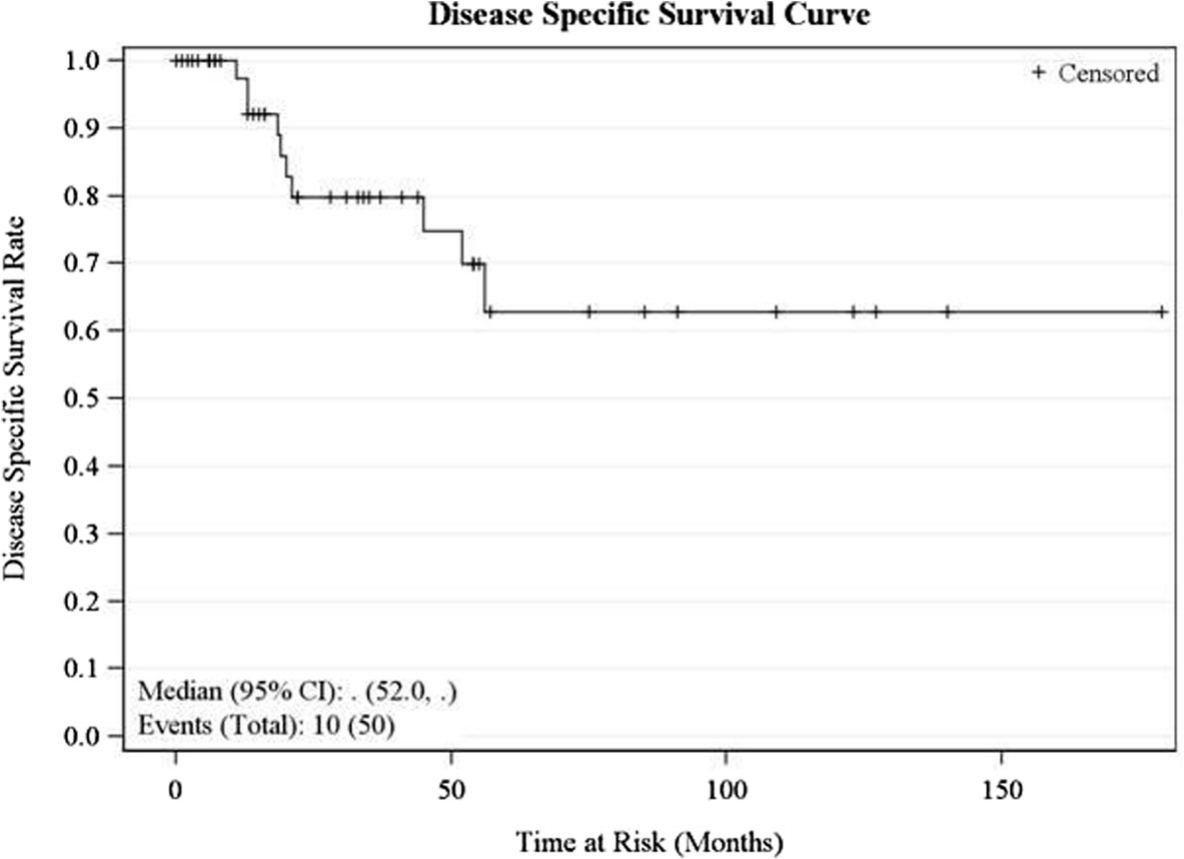
Disease-specific survival curve

**Fig. 3:**
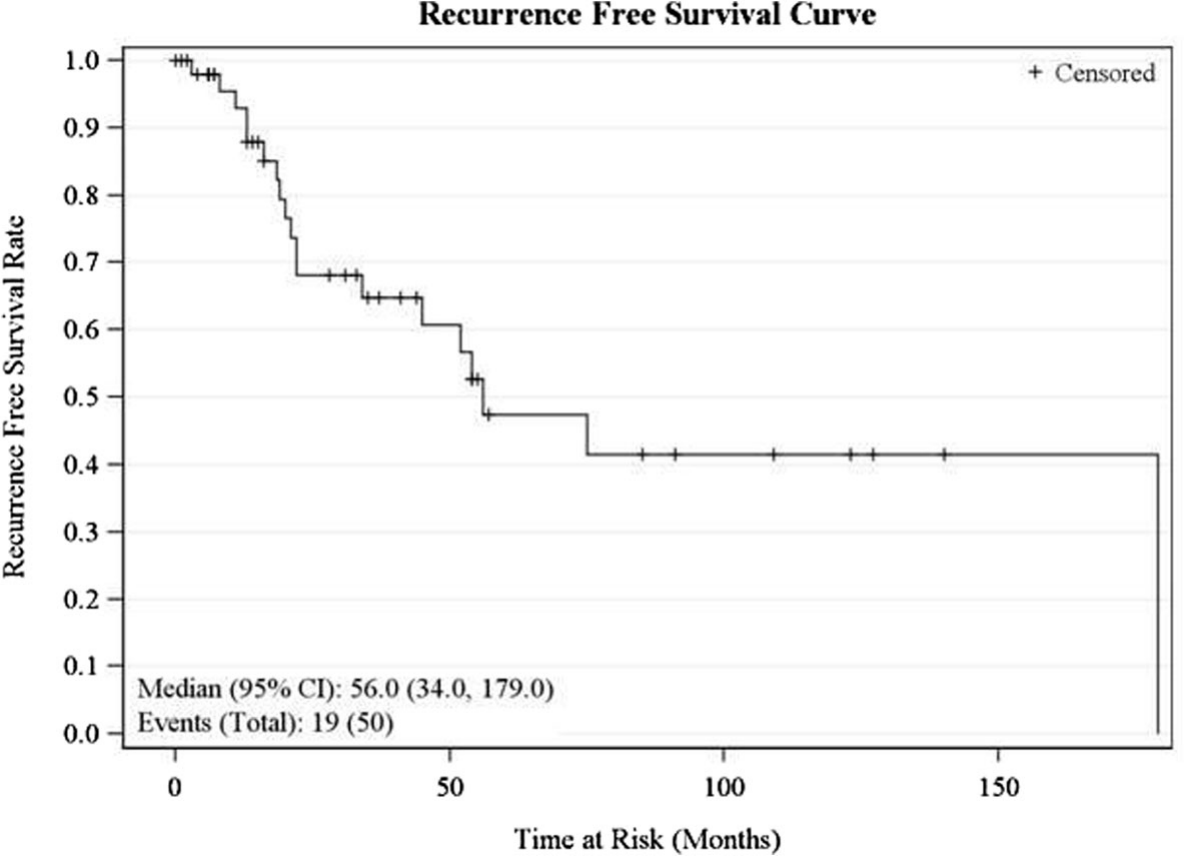
Recurrence-free survival curve

We determined overall survival, disease-specific survival, and recurrence-free survival for our series (Figs. 1, 2 and 3). Overall survival was defined as the time between the date of diagnosis and the date of death or the date of last follow-up or August 31, 2012. Disease-specific survival was defined as the time between the date of diagnosis and the date of death specifically from MATS, while recurrence-free survival was the time between initial treatment of disease and local, regional, or systemic recurrence of disease.

Overall, recurrence-free, and disease-specific survival analyses were done using the Kaplan-Meier method. Univariate and multivariate analyses were done, using Cox proportional hazards regression models, to determine predictors of overall survival, recurrence-free survival, and disease-specific survival. Variables used in the regression analyses include age, gender, primary site of disease, histologic subtype, type of surgical treatment, chemotherapy, radiation treatment, histologic grade, and TNM stage.

## Results

### Patient characteristics

Fifty patients were identified in this analysis. Fifty-six percent (28) of patients in our series were males, while 52% (26) were less than or 60 years old (Table 1). The median and mean ages for the series were 59.5 and 62.4 years, respectively.

**Table 1:** Demographics and univariate analysis

Patient characteristics	Number (%)	OS	DSS	RFS
Age				
≤ 60 years	26 (52)	0.001	0.89	0.02
> 60 years	24 (48)			
Sex				
Male	28 (56)	0.73	0.25	0.19
Female	22 (44)			
Primary site				
Head and neck	26 (52)			
Trunk	8 (16)	0.96	0.87	0.53
Upper extremities	8 (16)			
Lower extremities	8 (16)			
T stage				
T1	22 (44)	0.18	0.12	0.04
T2	9 (18)			
T3	1 (2)			
T4	5 (10)			
Tx	13 (26)			
N stage				
N0	44 (88)	0.15	0.01	0.03
N1	4 (8)			
N2	2 (4)			
M stage				
M0	30 (60)	0.54	0.19	0.16
M1	0 (0)			
Mx	20 (40)			
Histologic grade				
G1	5 (10)	0.92	0.85	0.85
G2	8 (16)			
G3	8 (16)			
Gx	29 (58)			
Perineural invasion				
Present	2 (4)	–	–	–
Absent	48 (96)			
Lymphovascular invasion				
Present	1 (2)	–	–	–
Absent	49 (98)			
Type of surgery				
Wide excision	15 (30)	0.47	0.39	0.17
Mohs surgery	11 (22)			
Local excision	23 (46)			
Chemotherapy				
Yes	4 (8)	0.40	0.02	0.13
No	46 (92)			
Radiation treatment				
Yes	7 (14)	0.42	0.06	0.47
No	43 (86)			
Recurrence				
Yes	6 (12)	0.83	–	–
No	40 (80)			
Unknown	4 (8)			
Type of recurrence				
Distant	2 (4)	0.98	–	–
Local	3 (6)			
Regional	1 (2)			

Limited observations in perineural invasion, lymphovascular invasion, and recurrence precluded their inclusion in the univariate analysis for OS, DSS, and RFS

### Tumor characteristics

Over half of the series (56%) involved the head and neck region (Table 1). The histology subtypes are outlined in Table 3. Perineural invasion and lymphovascular invasion were uncommonly observed at 4 and 2%, respectively (Table 1). Twenty-nine patients (58%) had undocumented histologic grades, while intermediate and poor grades of differentiation constituted 16% each. The remaining 10% were well differentiated (Table 1).

### Treatment

Altogether, 49 patients [98%] underwent surgery-wide local excision (30%), Moh’s micrographic surgery (22%), and simple excision (46%) (Table 1). One patient with skin appendage carcinoma involving the eyebrow was treated primarily with chemotherapy (carboplatin and taxol) followed by radiation (70 Gy). Four patients (8%) received chemotherapy. Three of whom were treated adjuvantly, while one was primary systemic chemotherapy. Out of the three which received adjuvant chemotherapy, one was treated with radiotherapy as well. Single-agent regimen was administered to two patients using cisplatin and paclitaxel, respectively. The remaining two patients received multiple-agent regimen. One was treated with carboplatin and paclitaxel, while the other received adriamycin, cytoxan, and paclitaxel. The indications for chemotherapy were nodal metastasis (2 patients), positive margin and perineural invasion (1 patient), and aggressive disease with periorbital involvement which would have necessitated extensive resection with orbital exenteration in an octogenarian. Seven patients had radiotherapy—two of whom also had chemotherapy (as indicated above). Out of these seven patients, six received radiation as adjuvant treatment while one received radiation as part of definitive chemoradiation. The indications for radiation were locally aggressive disease, perineural invasion, and nodal metastasis.

### Recurrence

The recurrence rate was 12%—three local recurrences, one regional recurrence, and two distant recurrences. Two of the local recurrences involved the lower extremity, while one occurred in the head and neck region. The distant recurrences were from head and neck primaries which metastasized to the groin and brain. The only regional occurrence was from a primary on the face which recurred in another area of the face. The pattern is such that two thirds of patients with recurrent disease had primary site in the head and neck region while the remaining one third had lower extremity as the primary site. The histologic subtypes for patients with recurrent disease include adenoid cystic carcinoma (2 patients), skin appendage carcinoma (2 patients), eccrine adenocarcinoma (1 patient), and sebaceous adenocarcinoma (1 patient). Time to recurrence ranged between 12.9 and 56 months, with the median time to recurrence of 20.3 months.

### Survival

The median overall survival for our series was 158 months (95% CI 52, 255 months). Five-year OS was 62.4% (95% CI 43.4, 76.6), while the 10-year OS was 56.7% (95% CI 36.4–72.7). Univariate analysis showed that advanced age (greater than 60 years) was a negative predictor (*P* = 0.001) of OS (Table 1). Both 5-year and 10-year disease-specific survival was 62.9% (95% CI 39.3–79.4). Nodal disease (*P* = 0.03) and treatment with chemotherapy (*P* = 0.02) were associated with worse disease-specific survival (DSS) on univariate analysis (Table 1). The recurrence-free survival rate was 47.4% (95% CI 28.2–64.4) at 5 years and 41.5% (95% CI 22.21–59.8) at 10 years. Age greater than 60 years (*P* = 0.02), advanced T stage (*P* = 0.04), and nodal disease (*P* = 0.03) were negative predictors of recurrence-free survival (RFS) on univariate analysis (Table 1).

On multivariate analysis, age greater than 60 years (*P* = 0.0008, HR = 12.9) was an independent negative predictor of overall survival. The presence of nodal disease (*P* = 0.03, HR = 7.2) and treatment with chemotherapy (*P* = 0.03, HR = 14.2) turned out as independent negative predictors of DSS. Similarly, advanced age (*P* = 0.0003, HR 12.5) and nodal disease (*P* = 0.04, HR 2.4) were independently predictive of higher incidence of recurrence on multivariate analysis (Table 2).

**Table 2:** Multivariate analysis for OS, DSS, and RFS

		HR (hazard ratio), 95% CI, and *P* value		
		OS	DSS	RFS
Patient characteristics				
Age	≤ 60 years	12.90 (2.87–57.95), 0.0008	–	12.53 (3.21–48.95), 0.003
	> 60 years			
Nodal metastasis		–	7.22 (0.722–72.186), 0.03	2.37 (0.22–25.05), 0.04
Chemotherapy		–	14.21 (1.27, − 158.91), 0.03	–

## Discussion

Previous studies have reported median ages ranging from 68 to 70 years [1, 4, 5]. The mean and median ages reported for our series were closer to those reported in a 48-patient series of microcystic adnexal carcinoma/sclerosing sweat duct carcinoma [6], which constituted 12% of histologic subtypes in our series (Table 3). Advanced age greater than 60 years was independently predictive of poor overall and recurrence-free survival in our study. This aligned well with the findings in other series where poor OS was observed on univariate analysis for patients with age greater than 70 years. Martinez et al. and Avraham et al. reported OS and DSS advantage with the female gender [1, 5], but gender was not predictive of OS, DSS, or RFS in our series. However, gender distribution showed male predominance (56%) in our study, a finding concordant with the Blake’s series [4] but in contrast with other studies [3, 7].

**Table 3:** Histologic subtypes

Histology	Number (%)
Skin appendage carcinoma	10 (20)
Eccrine adenocarcinoma	10 (20)
Sebaceous adenocarcinoma	9 (18)
Malignant eccrine poroma	6 (12)
Sclerosing sweat duct adenocarcinoma	6 (12)
Adenoid cystic carcinoma	5 (10)
Malignant eccrine spiradenoma	1 (2)
Malignant nodular hidradenoma	1 (2)
Porocarcinoma	1 (2)
Apocrine adenocarcinoma	1 (2)

Over half (52%) of the MATS in our series were located in the head and neck region. This is consistent with most series [1, 4, 5]. The remaining anatomic sites (upper extremities, lower extremities, and trunk) had equal distribution of 16% each. The vast majority had early T stage disease, with 44% being T1. The proportion of unknown T stage (Tx) in our series was about half of those in two large population-based series which reported Tx in the range of 46–56% [1, 4]. Advanced T stage was a negative predictor of recurrence-free survival on univariate analysis (*P* = 0.04), but this trend failed to persist on multivariate analysis. Unlike squamous cell carcinoma of the skin and melanoma [8, 9], there was no association between T stage and nodal metastasis. No patient in our series had distant metastasis on presentation, although as many as 40% were documented as unknown M stage. Two distant recurrences were documented for adenoid cystic carcinoma and skin appendage carcinoma, with primaries in the head and neck region. Distant metastases were recorded in the literature for nodular hidradenocarcinoma, eccrine porocarcinoma, apocrine carcinoma [10], and microcystic adnexal carcinoma [11]. The histologic grade of the tumors was not predictive of OS, DSS, and RFS on univariate and multivariate analysis in our series. Caution, however, must be exercised as 58% of patients did not have documented histologic grade. This is a reflection of how pathology reporting system for this group of tumors has evolved over the years, with grade reported for the more recent cases. This trend was similarly observed in other series, with undocumented histologic grades in the range of 76 to 81% of patients [1, 5]. A study reported survival advantage for well-differentiated tumors on univariate analysis, but this variable was not predictive on multivariate analysis [5]. Another study also demonstrated survival advantage with better histologic grades, albeit after excluding patients with distant metastasis [1]. We did not identify any histologic subtype with survival advantage in our analysis. The existing literature, however, showed a mixed picture, with some reporting an advantage for microcystic adnexal carcinoma [5], while other studies favored sebaceous adenocarcinoma [4, 12] or apocrine adenocarcinoma [1].

Surgical nodal staging was done for 12% of the patients in our series. Histopathologic nodal evaluation varied from 11 to 29% in the literature. There were no standardized criteria for selecting patients who required nodal sampling. Sixty-six percent (4 out of 6) of patients who had nodal basin evaluation in our series underwent the procedure because of clinically positive lymph nodes. One patient had sentinel lymph node biopsy done based on surgeon’s clinical decision, while the sixth patient had the procedure done due to unfavorable histologic criteria (poor differentiation and lymphovascular invasion). In a similarly sized series of 48 patients, nodal sampling was done for nine patients (18.8%) who developed local recurrence [13]. Four out of these nine patients demonstrated nodal metastasis. This group of researchers advocated for nodal sampling in patients with recurrent disease who presumably were preselected by their aggressive biology. On the other hand, Ogata et al., in a series of nine patients with apocrine carcinoma who had wide local excision and routine regional lymph node dissection, showed nodal disease in all but one patient [14]. This group called for routine nodal staging, at least for apocrine carcinoma. Experience from breast cancer and melanoma has shown that nodal metastasis can be present in the absence of clinically positive lymph nodes. Since nodal basin is grossly under evaluated, we do not have accurate information yet on incidence of nodal metastasis and its effect on recurrence and survival. It is worthwhile to evaluate MATS population with nodal metastasis with a view to determine predictors of nodal metastasis and then prospectively validate identified predictors. Prospective validation requires a larger cohort of patients which is always a challenge when addressing key issues on these rare tumors. Same could be said to apply to histologic criteria like grade, perineural invasion, and angiolymphatic invasion. These have been shown to be important in prognostication for melanoma and many gastrointestinal cancers. If validated, they should be incorporated into the staging system which means pathologists would report these features. Due to limited observations in these categories, we did not include them in our survival analysis. Only 4% of patients in our series were positive for perineural invasion and 2% for lymphovascular invasion. We observed most studies on MATS did not address these two important criteria.

The role of adjuvant radiation and chemotherapy is not well defined for MATS. To address this, we need a combination of large study population and details on regimen of treatment. Previous studies with much lower number of patients than our series had reported on adjuvant chemoradiation. The large population-based series from SEER database were limited, as there was no information on chemotherapy while radiation treatment was documented as a categorical variable without detailed information on selection criteria and dose. Unfortunately, for rare and heterogeneous tumors like MATS, this will always be challenging. Current proposals on the role of adjuvant radiation support the use of postoperative radiotherapy for cases in which sufficient resection margins cannot be achieved because of the anatomic site of the lesion or with positive resection margins [14, 15]. There are no defined guidelines/protocols for adjuvant chemotherapy in the management of MATS, but there are reported cases of recurrent or metastatic diseases treated with chemotherapeutic and targeted agents [16]. Various chemotherapeutic agents like doxorubicin, mitomycin, vincristine, 5-fluorouracil, cyclophosphamide, anthracycline, bleomycin, paclitaxel, cisplatin, and carboplatin were used in different combinations for metastatic disease [17, 18]. Results varied from no response to stable disease and partial response. This trend was noticed in all four patients (8%) who received chemotherapy in our series. The histologic subtypes represented in this subgroup were adenoid cystic carcinoma, eccrine adenocarcinoma, apocrine adenocarcinoma, and skin appendage carcinoma. Chemotherapeutic agents utilized were cisplatin, carboplatin, adriamycin, cytoxan, and paclitaxel. Drawing inference from the apocrine-eccrine origin of many of these tumors, some proponents have made a case for treatment with chemotherapy regimen used for breast cancer. For our series, 4% received adjuvant chemotherapy alone, 10% were treated with adjuvant radiation alone, and 4% received adjuvant chemoradiation. The survival analysis showed poor RFS for patients treated with chemotherapy. These patients may have been preselected by the aggressive biology of their tumors. The role of chemotherapy in these patients needs further study. Treatment with radiation, while not associated with poor survival outcome, did not translate to survival advantage either.

Six patients (12%) had recurrent disease in this series. There were three local recurrences, one regional recurrence and two distant recurrences. Four histologic subtypes were represented in this subgroup: sebaceous adenocarcinoma (1 regional recurrence), eccrine adenocarcinoma (1 local recurrence), adenoid cystic carcinoma (1 local and 1 distant recurrence), and skin appendage carcinoma (1 local and 1 distant recurrence). Four of the patients with recurrent disease had their primary lesions located on the head and neck region while the remaining two were located on the lower extremities. Recurrence-free survival analysis was done and showed median RFS of 56 months. Five-year and 10-year RFS were 47.4% (95% CI 28.2–64.4) and 41.5% (95% CI 22.21–59.8), respectively. Univariate analysis showed age greater than 60 years, positive nodal status, and advanced T stage as predictors of RFS, but only age and positive nodal status persisted as independent predictors of RFS on multivariate analysis. Data on recurrence pattern is crucial to patient’s education about the prognosis of these tumors. There is paucity of similar data in the literature.

## Conclusion

So far, there are few large population-based studies available on malignant adnexal tumors of the skin (MATS). Most of these were derived from the SEER database [1, 4, 5]. These studies had the benefits of large study population and broader representation of the population at large. They were, however, not without their shortcomings which included lack of uniform pathology reporting, absence of detailed information about margin status, recurrences, and selection criteria for nodal sampling, adjuvant chemotherapy, and radiation treatment. We reviewed our 50-patient, single-institution series and were able to address some of these limitations, albeit with limited numbers.

This study shows that younger patients had better OS and RFS. Absence of nodal metastasis was also noted to translate to better RFS and DSS. Lymph node basin staging is worth considering in the workup and treatment. More importantly, strategies that promote early detection and prompt treatment should be emphasized in addressing this disease.

## Abbreviations

DSS: Disease-specific survival
MATS: Malignant adnexal tumors of the skin
OS: Overall survival
RFS: Recurrence-free survival
